# Case Report: Transient myocardial thickening in a cat secondary to acute cholangiohepatitis

**DOI:** 10.3389/fvets.2025.1703872

**Published:** 2026-01-16

**Authors:** Byung-Jun Kim, Mi-Kyung Park, Kun-Ho Song

**Affiliations:** 1 College of Veterinary Medicine, Chungnam National University, Daejeon, Republic of Korea; 2 Hannam Cardiology and Internal Medicine Animal Hospital, Seoul, Republic of Korea; 3 Research Institute of Veterinary Medicine, Chungnam National University, Daejeon, Republic of Korea

**Keywords:** cat, transient myocardial thickening, acute cholangiohepatitis, cardiac troponin I, N-terminal pro-B-type natriuretic peptide

## Abstract

Transient myocardial thickening (TMT) has been reported mainly in young cats following systemic triggers such as anesthesia, surgery, acute stress, or infection; however, to the authors’ knowledge, TMT secondary to acute cholangiohepatitis has not been described. A 3-year-old, 5.8-kg castrated male Abyssinian was referred with acute cholangiohepatitis. Initial evaluation revealed increased hepatic enzymes and bilirubin, elevated cardiac troponin I (cTnI, 3.5 ng/mL), and mildly increased N-terminal pro-B-type natriuretic peptide (NT-proBNP, 102.6 pmol/L). On the day of discharge, despite improving hepatic indices, cTnI rose abruptly to 8.0 ng/mL and NT-proBNP exceeded 1,500 pmol/L. Echocardiography demonstrated septal thickening, left atrial enlargement, and systolic anterior motion of the mitral valve, consistent with TMT. Atenolol was added to the outpatient medical management for cholangiohepatitis, consisting of broad-spectrum antimicrobials, hepatoprotectants, and antiemetic/gastroprotective agents. Over the course of 84 days, five follow-up evaluations were performed, during which hepatic values normalized, cTnI and NT-proBNP returned to reference ranges, and myocardial dimensions and vertebral heart score normalized. Unlike most reports in which TMT is identified after congestive signs develop, this case was recognized earlier, on the basis of an abrupt biomarker surge during apparent clinical improvement.

## Introduction

Transient myocardial thickening (TMT) is a reversible myocardial disorder reported predominantly in young cats (1–3). Its initial echocardiographic features often resemble hypertrophic cardiomyopathy (HCM), but a defining characteristic of TMT is subsequent resolution of wall thickening, in contrast to the irreversible remodeling of HCM (2, 3). Many cases are diagnosed only after the onset of congestive signs such as pulmonary edema, pleural effusion or dyspnea, making early recognition challenging (1, 2).

Reported triggers of TMT include anesthesia, surgery, systemic inflammation or infection, and acute stress, with secondary catecholamine surges considered part of the stress response (1, 4–6). Proposed mechanisms involve transient myocardial edema and sympathetic overactivation (7, 8). Diagnosis relies on serial echocardiography with cardiac biomarkers, most notably cardiac troponin I (cTnI), which directly reflects acute cardiomyocyte injury (1, 2, 8, 9). Management focuses on treating the underlying cause; standard heart failure therapy is applied when indicated, and β-blockers such as atenolol may be considered in cases with left ventricular outflow tract obstruction (LVOTO), often associated with systolic anterior motion (SAM) of the mitral valve and a turbulent outflow jet on Doppler (2, 8, 10, 11).

Acute cholangiohepatitis is an inflammatory hepatobiliary disease in cats, characterized by fever, vomiting, inappetence, lethargy, and jaundice, with laboratory evidence of increased hepatic enzymes and hyperbilirubinemia (12–14). Supportive treatment is primary, with antibiotics and adjunctive medications indicated when bacterial infection is suspected (13, 14).

To the authors’ knowledge, this is the first report of TMT secondary to acute cholangiohepatitis in a cat. The diagnosis was prompted by a marked surge in cTnI during clinical improvement and was confirmed by serial echocardiography and biomarker normalization.

## Case description

A 3-year-old, 5.8-kg castrated male Abyssinian cat was initially examined at a local veterinary clinic for eight episodes of vomiting, fever, inappetence, and lethargy. Because hepatobiliary disease was suspected, the patient was referred to a 24-h hospital. Abdominal ultrasonography revealed gallbladder wall 1.1 mm (reference ≤ 1.0 mm), bile duct diameter 3.0 mm (reference ≤ 4.0 mm), and increased parenchymal echogenicity. A rectal temperature of 40.1 °C (reference interval 38.0–39.2 °C) was recorded, and a diagnosis of acute cholangiohepatitis was made. Supportive treatment, including fluids and antimicrobials, was administered for 3 days but clinical improvement was limited; the patient was subsequently referred to our hospital for continued management and advanced cardiac assessment.

On presentation to our hospital, the cat remained lethargic and hyporexic. Heart rate was 180 beats/min (reference 140–220 beats/min) without an auscultable murmur; rectal temperature was 39.9 °C, consistent with persistent fever; and systolic blood pressure measured by Doppler was 130 mmHg (ACVIM normal 90–139 mmHg). Serum biochemistry revealed alkaline phosphatase (ALKP) 408 U/L (reference interval, 38–165 U/L), alanine aminotransferase (ALT) > 1,000 U/L and aspartate aminotransferase (AST) > 1,000 U/L (upper reporting limit 1,000 U/L for both on the FUJIFILM DRI-CHEM NX600), and total bilirubin 4.8 mg/dL (reference interval, 0.1–0.4 mg/dL). Serum chemistry analytes were measured on the FUJIFILM DRI-CHEM NX600 (FUJIFILM Corp., Tokyo, Japan). Cardiac biomarkers were measured on the BIONOTE Vcheck platform (BIONOTE, Inc., Hwaseong-si, Gyeonggi-do, Republic of Korea). N-terminal pro-B-type natriuretic peptide (NT-proBNP) was 102.6 pmol/L (reference interval, 0–100 pmol/L) and cTnI was 3.5 ng/mL (reference interval, 0–0.18 ng/mL). Thoracic radiography revealed a vertebral heart score (VHS) of 7.7 (reference interval 6.7–8.1), which was within the normal range.

Echocardiography demonstrated mild septal thickening (interventricular septal thickness in diastole (IVSd) 6.2 mm (reference < 5.5 mm), a left atrium-to-aorta ratio (LA/Ao) of 1.20 (reference < 1.5), and a left atrial diameter (LAD) of 11 mm (measured on the right parasternal short-axis view at end-systole; reference ≤ 13 mm), without SAM. Differential diagnoses included early HCM, TMT, and pseudo-hypertrophy. Hospital treatment included balanced crystalloid fluids (Plasma Solution A) administered intravenously at 2 mL/kg/h; antimicrobials (ampicillin–sulbactam 30 mg/kg IV TID, marbofloxacin 3 mg/kg IV SID, metronidazole 15 mg/kg IV BID); hepatoprotectants (S-adenosyl-L-methionine 10 mg/kg PO BID, ursodeoxycholic acid 10 mg/kg PO BID, pentoxifylline 10 mg/kg PO BID); an antiemetic (maropitant 1 mg/kg SC SID); an anti-nausea agent (ondansetron 0.5 mg/kg IV BID); and a gastroprotectant (esomeprazole 1 mg/kg IV BID). Nutritional support was provided via a nasoesophageal tube with Royal Canin Recovery Liquid. Hepatic parameters (ALT, AST, ALP, and total bilirubin) began to improve during hospitalization (Table 1). Clinical signs gradually improved and discharge was planned.

**Table 1:** Timeline of clinical course, cardiac biomarkers, hepatic enzyme activities, radiographic indices, and echocardiographic parameters in a 3-year-old Abyssinian cat with transient myocardial thickening (TMT) secondary to acute cholangiohepatitis.

Parameter	Day 0 (admission)	Day 3 (discharge)	Day 13	Day 35	Day 84
Key events/therapy	Acute cholangiohepatitis diagnosed; hospitalization	Clinical improvement; atenolol initiated	1st follow-up atenolol continued	2nd follow-up atenolol discontinued	Final follow-up
cTnI (ng/mL)	3.5	8.0	0.61	0.01	0.01
NT-proBNP (pmol/L)	102.6	>1,500	407.6	132.3	<50
IVSd (mm)	6.2	8.6	7.4	6.8	5.4
LA/Ao	1.2	1.56	1.44	1.33	1.22
LAD (mm)	11	14	13	12	11
VHS	7.7	8.3	8.0	7.9	7.7
SAM	−	+	+	±	−
ALKP (U/L)	408	321	168	97	99
GGT (U/L)	12	4	8	8	5
ALT (U/L)	>1,000	>1,000	338	93	81
AST (U/L)	>1,000	559	112	34	32
T-BIL (mg/dL)	4.8	0.8	0.2	0.1	0.1

Cardiac troponin I (cTnI) and NT-proBNP increased markedly at discharge, coinciding with transient interventricular septal thickening and systolic anterior motion (SAM), followed by progressive normalization of biomarker values, liver enzyme activities, vertebral heart score (VHS), and echocardiographic measurements during serial follow-up. cTnI, cardiac troponin I; NT-proBNP, N-terminal pro-B-type natriuretic peptide; IVSd, interventricular septal thickness in diastole; LA/Ao, left atrium-to-aorta ratio; LAD, left atrial diameter (measured on the right parasternal short-axis view at end-systole); VHS, vertebral heart score; SAM, systolic anterior motion of the mitral valve; ALKP, alkaline phosphatase; GGT, gamma-glutamyl transferase; ALT, alanine aminotransferase; AST, aspartate aminotransferase; T-BIL, Total bilirubin.

At discharge re-evaluation, hepatic parameters had continued to improve (Table 1). However, cTnI had increased to 8.0 ng/mL (reference interval, 0–0.18 ng/mL) and NT-proBNP exceeded 1,500 pmol/L (reference interval, 0–100 pmol/L). VHS was 8.3 (reference interval 6.7–8.1), and echocardiography revealed IVSd 8.6 mm (reference < 5.5 mm), LA/Ao 1.56 (reference < 1.5), LAD 14 mm (reference ≤ 13 mm), and SAM of the mitral valve with dynamic LVOT obstruction (continuous-wave Doppler LVOT Vmax 5.14 m/s and peak pressure gradient 106 mmHg). These changes were consistent with TMT, and atenolol (6.25 mg/cat PO BID) was added to the outpatient cholangiohepatitis regimen consisting of amoxicillin–clavulanate (13.75 mg/kg PO BID), marbofloxacin (2.2 mg/kg PO BID), metronidazole (15 mg/kg PO BID); hepatoprotectants—S-adenosyl-L-methionine (10 mg/kg PO BID), ursodeoxycholic acid (10 mg/kg PO BID), and pentoxifylline (10 mg/kg PO BID); an antiemetic—maropitant (1 mg/kg PO SID); an anti-nausea agent—ondansetron (0.5 mg/kg PO BID); and a gastroprotectant—esomeprazole (1 mg/kg PO BID).

At the first follow-up, biochemical evidence of cholangiohepatitis was improving further. Cardiac biomarkers changed in parallel; cTnI had decreased to 0.61 ng/mL (reference interval, 0–0.18 ng/mL) and NT-proBNP to 407.6 pmol/L (reference interval, 0–100 pmol/L). VHS was 8.0 (reference interval 6.7–8.1), IVSd 7.4 mm (reference < 5.5 mm), LA/Ao 1.44 (reference < 1.5), and LAD 13 mm (reference ≤ 13 mm). SAM persisted, and atenolol was continued. By the second follow-up, hepatic values were near normal, cTnI had normalized to 0.01 ng/mL (reference interval, 0–0.18 ng/mL), and NT-proBNP had decreased to 132.3 pmol/L (reference interval, 0–100 pmol/L). VHS was 7.9 (reference interval 6.7–8.1), IVSd 6.8 mm (reference < 5.5 mm), LA/Ao 1.33 (reference < 1.5), and LAD 12 mm (reference ≤ 13 mm). Atenolol was discontinued at this time.

**Figure 1:**
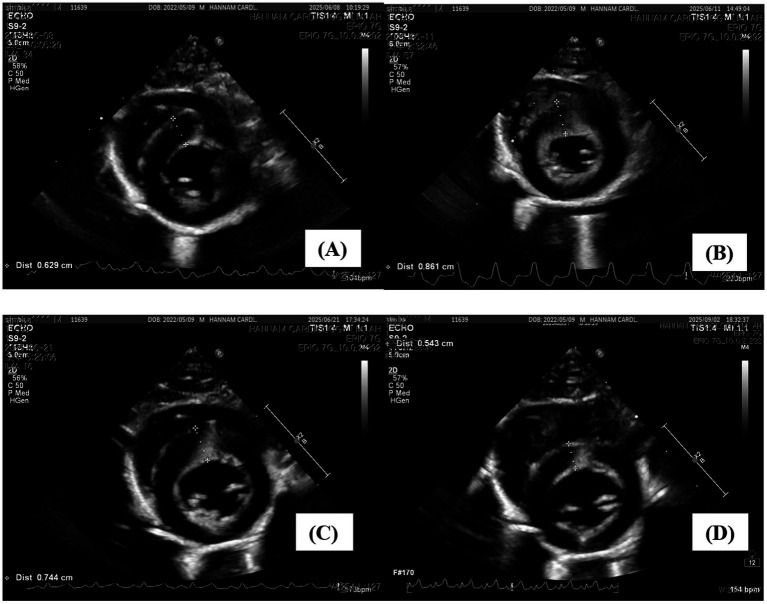
Serial short-axis echocardiographic images demonstrating changes in interventricular septal thickness over time. **(A)** Day 0 (admission): IVSd 6.2 mm. **(B)** Day 3 (discharge): IVSd 8.6 mm. **(C)** Day 13 (1^st^ follow-up): IVSd 7.4 mm. **(D)** Day 84 (final follow-up): IVSd 5.4 mm. IVSd, interventricular septal thickness in diastole.

**Figure 2:**
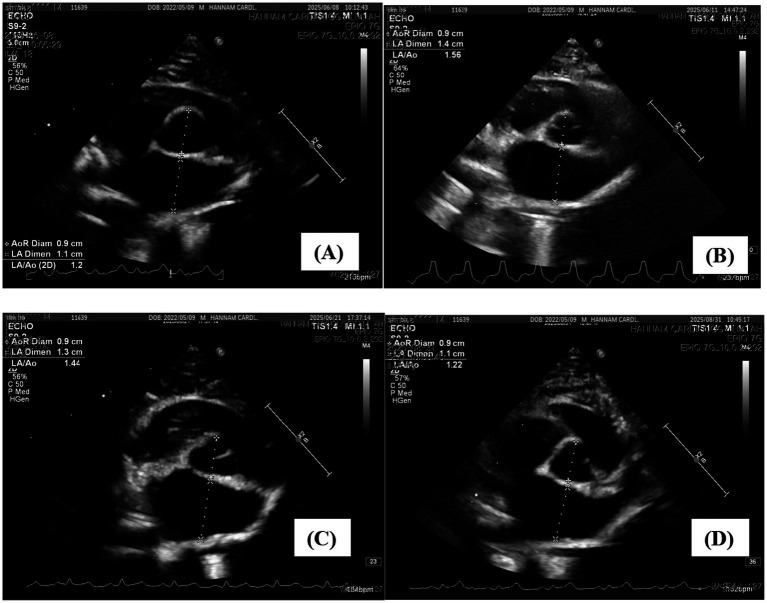
Serial right parasternal short-axis echocardiographic images showing changes in left atrial size (LA/Ao ratio). **(A)** Day 0 (admission): LA/Ao 1.2. **(B)** Day 3 (discharge): LA/Ao 1.56. **(C)** Day 13 (1^st^ follow-up): LA/Ao 1.44. **(D)** Day 84 (final follow-up): LA/Ao 1.22. LA/Ao, left atrium-to-aorta ratio.

**Figure 3:**
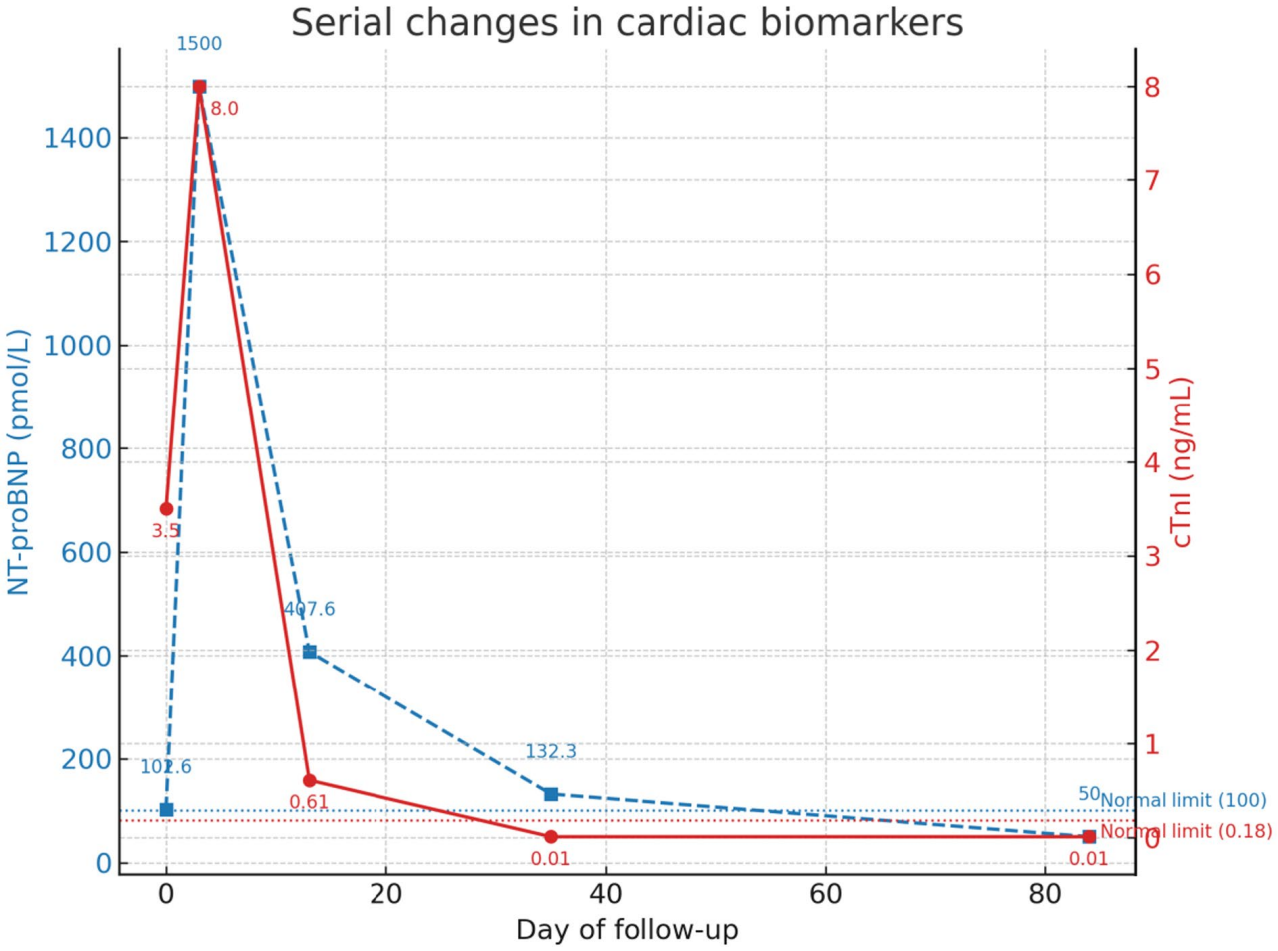
Serial changes in cardiac biomarkers over 84 days. Temporal trends in cardiac troponin I (cTnI, red solid line, right y-axis) and N-terminal pro-B-type natriuretic peptide (NT-proBNP, blue dashed line, left y-axis) measured at five time points: Day 0 (admission), Day 3 (discharge), Day 13, Day 35, and Day 84 (final follow-up). Peak values were observed at discharge (cTnI 8.0 ng/mL; NT-proBNP >1,500 pmol/L), followed by progressive decline to within reference limits (cTnI <0.18 ng/mL; NT-proBNP <100 pmol/L) by Day 84. Dashed horizontal lines indicate the upper reference limits for NT-proBNP (100 pmol/L) and cTnI (0.18 ng/mL).

At the final follow-up, hepatic enzymes and bilirubin were within reference intervals (Table 1); cardiac biomarkers were likewise normal, with cTnI <0.01 ng/mL (reference interval 0–0.18 ng/mL) and NT-proBNP <50 pmol/L (reference interval 0–100 pmol/L). Thoracic radiography showed VHS 7.7 (reference interval 6.7–8.1), and echocardiography demonstrated IVSd 5.4 mm (reference < 5.5 mm), LA/Ao 1.22 (reference < 1.5), and LAD 11 mm (reference ≤ 13 mm), with complete resolution of SAM. The cat was clinically normal and remained healthy at the time of reporting. Serial trajectories of septal thickness (IVSd), left atrial size (LA/Ao), and cardiac biomarkers (cTnI, NT-proBNP) are shown in Figures 1–3 (Figures 1, 2 illustrate echocardiographic indices at four time points—panels A–D correspond to Day 0 [admission], Day 3 [discharge], Day 13, and Day 84—whereas Figure 3 presents biomarker trends across all five assessments, including Day 35).

## Discussion

To the authors’ knowledge, this is the first report of transient myocardial thickening (TMT) secondary to acute cholangiohepatitis in a young cat, identified prior to the onset of cardiogenic pulmonary edema, pleural effusion or severe dyspnea on the basis of an abrupt biomarker surge. This case is notable for documenting both structural and biochemical reversibility across five serial assessments over an 84-day period. Prior publications indicate that TMT is over-represented in young cats and initially overlaps echocardiographically with hypertrophic cardiomyopathy (HCM); definitive distinction typically requires serial echocardiography demonstrating normalization of myocardial wall thickness and left atrial (LA) size over time (1–3). Moreover, cTnI elevation is commonly observed at presentation, whereas BNP/NT-proBNP responses are more variable and are best regarded as supportive rather than determinative (2, 6, 8–10, 15). The present case conforms to these features (young age, marked cTnI rise, early overlap with HCM, and documented reversibility) and uniquely captures a paradoxical biomarker surge during clinical improvement, prompting timely cardiac reassessment. Importantly, normalization of cardiac parameters occurred in parallel with the resolution of hepatobiliary inflammation (Table 1; Figures 1–3).

A major strength of this report is the structured, multi-modal follow-up, with concurrent measurements of cTnI, NT-proBNP, thoracic radiography (vertebral heart score, VHS), and echocardiography (interventricular septal thickness in diastole [IVSd], LA-to-aorta ratio [LA/Ao], LA diameter, and presence of systolic anterior motion [SAM]) at five time points (illustrated in Figures 1–3). In addition to the stepwise normalization of septal thickness, LA size, and VHS, hepatic biochemical indices improved over the same intervals, underscoring clinicopathologic coupling between the inflammatory trigger and myocardial recovery (see Table 1). On the day of discharge, a sharp rise in cTnI accompanied by an increase in NT-proBNP coincided with new septal thickening and SAM; short-term atenolol, together with continued treatment of the precipitating disease, was followed by concordant normalization of biomarkers and imaging indices, exemplifying the reversible nature of TMT. In the literature, resolution of TMT is typically achieved over weeks to months (median ~43 days) (2, 3); in the present case, both echocardiographic indices and biomarkers normalized by Day 84, a timeframe within the reported range, albeit longer than the median.

The pathophysiologic paradigm for TMT implicates acute systemic inflammation and sympathetic overactivation, resulting in transient myocardial edema, microcirculatory disturbance, and altered wall stress (1, 6–8, 16, 17). Increased sarcolemmal permeability plausibly accounts for cTnI leakage, whereas attenuation of the systemic inflammatory burden permits recovery of myocardial structure and biomarker values (1–3, 6–9). The clinical sequence observed here—fever and cholestatic enzyme elevation → abrupt cTnI surge with IVSd/SAM appearance → parallel normalization of cTnI, NT-proBNP, and structural indices as hepatobiliary disease resolved—aligns with this model.

From a biomarker standpoint, cTnI is the most sensitive and direct indicator of acute cardiomyocyte injury in TMT, exhibiting the greatest dynamic range and earliest signal (2, 8, 9). By contrast, NT-proBNP, a marker of wall stress, is more variable across individuals and clinical contexts and is best interpreted as adjunctive (10, 15, 18, 19). In this cat, the cTnI spike followed by normalization mirrored the clinical and imaging course, whereas NT-proBNP rose and then declined into reference limits, reinforcing their complementary roles with cTnI as the primary marker for detection and monitoring.

Differential diagnosis warrants particular attention. TMT can mimic HCM with septal hypertrophy, LA enlargement, and SAM/left ventricular outflow tract obstruction (LVOTO) at a single examination (2, 3, 8, 15); however, normalization of wall thickness and LA size within weeks to months is a hallmark of TMT and contrasts with the irreversible remodeling characteristic of HCM (10, 15). Dehydration-related pseudo-hypertrophy should also be considered (20, 21); yet, in this case wall thickening and biomarker escalation persisted despite appropriate fluid and nutritional support, resolving only as the underlying inflammatory condition improved—findings that support a diagnosis of TMT. In addition, other potential cardiac rule-outs were considered, including conditions that could produce secondary hepatobiliary abnormalities through systemic venous congestion (e.g., right-sided heart failure due to tricuspid valve disease, pulmonary hypertension with right ventricular dysfunction, restrictive cardiomyopathy with predominant right-sided involvement, arrhythmogenic right ventricular disease, and pericardial disease). In this cat, echocardiography revealed no right-sided structural or hemodynamic abnormalities, and there were no clinical or imaging findings suggestive of congestive hepatopathy; therefore, hepatic enzyme elevations and hyperbilirubinemia were attributed to primary cholangiohepatitis rather than right-sided cardiac disease. This structured exclusion of right-sided etiologies, together with the documented reversibility of myocardial thickening and LA size, supports TMT as the most plausible diagnosis.

This report has limitations. First, histopathology or cardiac MRI was not performed, precluding definitive exclusion of primary cardiomyopathy; nonetheless, normalization of wall thickness and LA enlargement by Day 84 argues strongly against HCM. Second, we did not perform targeted infectious-disease testing for recognized precipitants of TMT—Bartonella spp., Toxoplasma gondii, and Mycoplasma spp.—because clinicopathologic data and treatment response strongly supported acute cholangiohepatitis as the primary trigger; accordingly, a concurrent infectious contribution cannot be entirely excluded. Third, β-blocker use in TMT is not standardized; atenolol was instituted specifically for severe dynamic LVOT obstruction associated with SAM (continuous-wave Doppler LVOT Vmax 5.14 m/s; peak pressure gradient 106 mmHg) and was discontinued as myocardial indices normalized. Prospective studies are needed to clarify indications, dosing, and duration of β-blockade in feline TMT.

This case highlights several important clinical implications. Acute systemic inflammatory diseases—particularly cholangiohepatitis—should be recognized as potential precipitants of TMT in cats. When cardiac biomarkers are increased in cats with hepatobiliary inflammation, we recommend integrating serial hepatic panels (ALT, AST, ALP, bilirubin) into the monitoring plan alongside prompt cardiac evaluation. Because TMT reflects acute myocardial injury and transient thickening, cTnI is the most sensitive and dynamically responsive marker, whereas NT-proBNP is best interpreted as supportive. Notably, an abrupt rise in cTnI—even during apparent clinical improvement—should trigger echocardiography and a full cardiac work-up. Finally, because TMT can mimic HCM on a single examination, documenting reversibility—normalization of myocardial wall thickness and LA size on serial echocardiography together with the return of cTnI and NT-proBNP to reference intervals—in parallel with biochemical resolution of hepatobiliary disease (Table 1) is decisive for distinguishing TMT from HCM and from dehydration-related pseudo-hypertrophy.

## Conclusion

This case provides the first evidence that acute cholangiohepatitis can serve as a novel systemic trigger for transient myocardial thickening in cats. Abrupt increases in cTnI and NT-proBNP may occur even during apparent clinical improvement, with cTnI functioning as the most sensitive biomarker for early recognition of myocardial injury. When such elevations are identified in cats with hepatobiliary disease, comprehensive cardiac assessment—including echocardiography—should be promptly undertaken. Furthermore, serial follow-up documenting both biomarker normalization and structural reversibility is decisive for distinguishing TMT from hypertrophic cardiomyopathy.

## Data Availability

The original contributions presented in the study are included in the article/Supplementary material, further inquiries can be directed to the corresponding author.
