# Supplementary figures and images for: Case Report: Transient myocardial thickening in a cat secondary to acute cholangiohepatitis
Source: Front Vet Sci. 2026 Jan 16;12:1703872. doi: 10.3389/fvets.2025.1703872 (PMC12855061; doi:10.3389/fvets.2025.1703872)

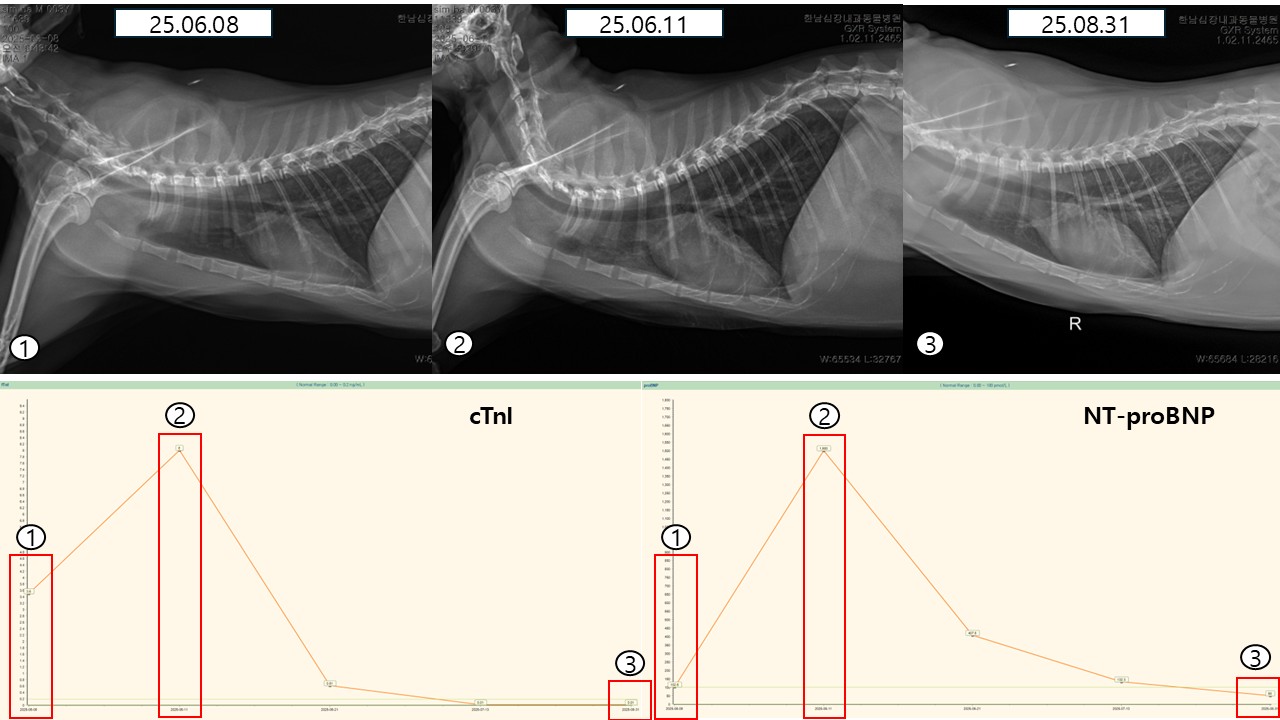

Supplement: Supplementary file 1 [file Image_1.jpeg]

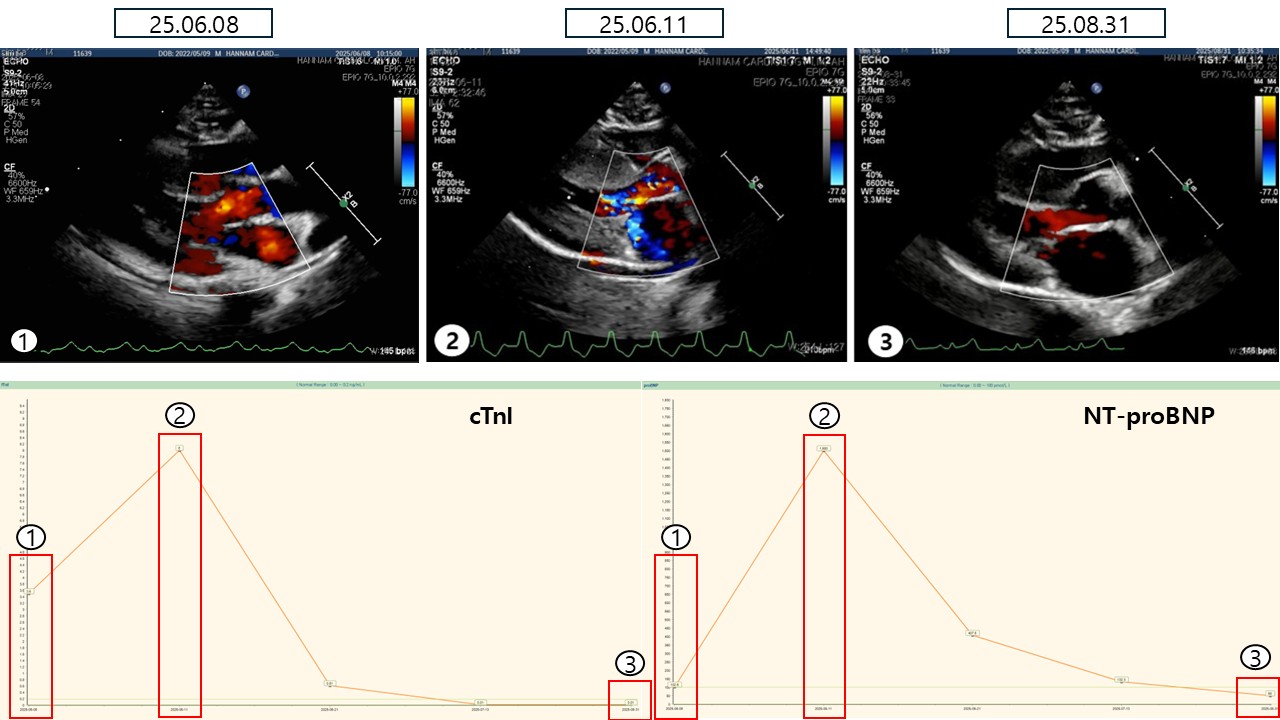

Supplement: Supplementary file 2 [file Image_2.jpeg]

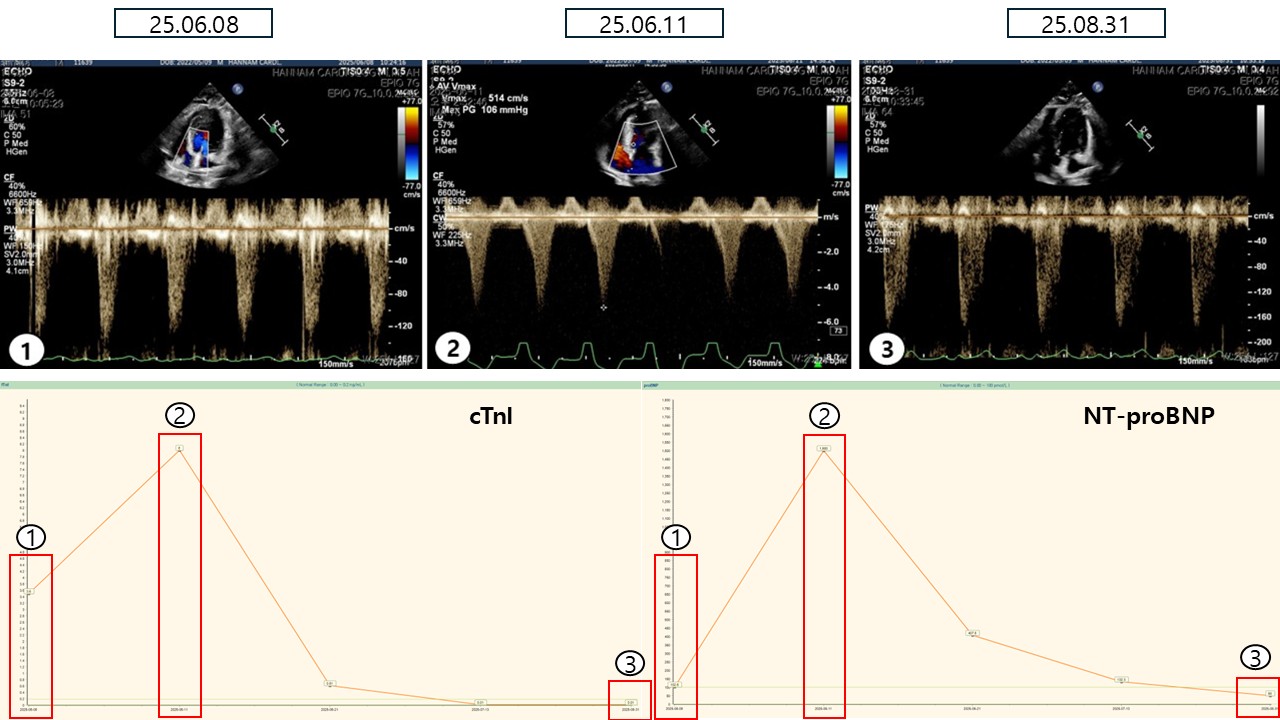

Supplement: Supplementary file 3 [file Image_3.jpeg]

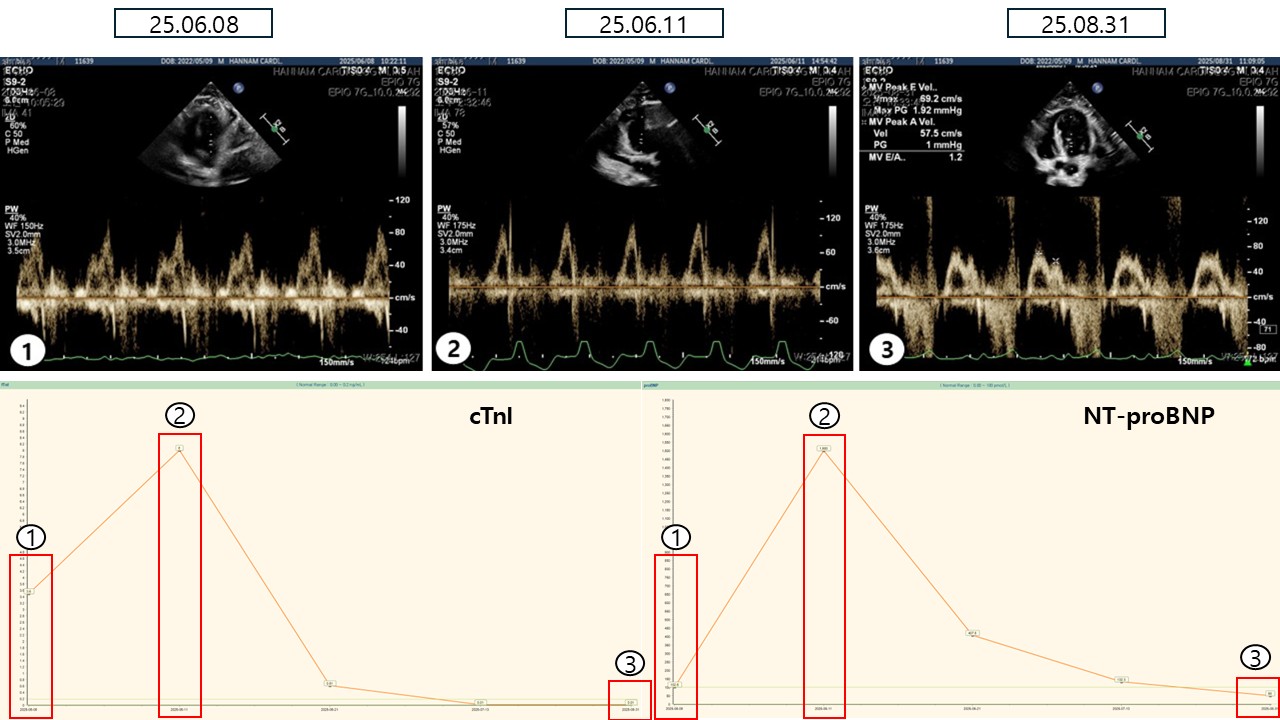

Supplement: Supplementary file 4 [file Image_4.jpeg]

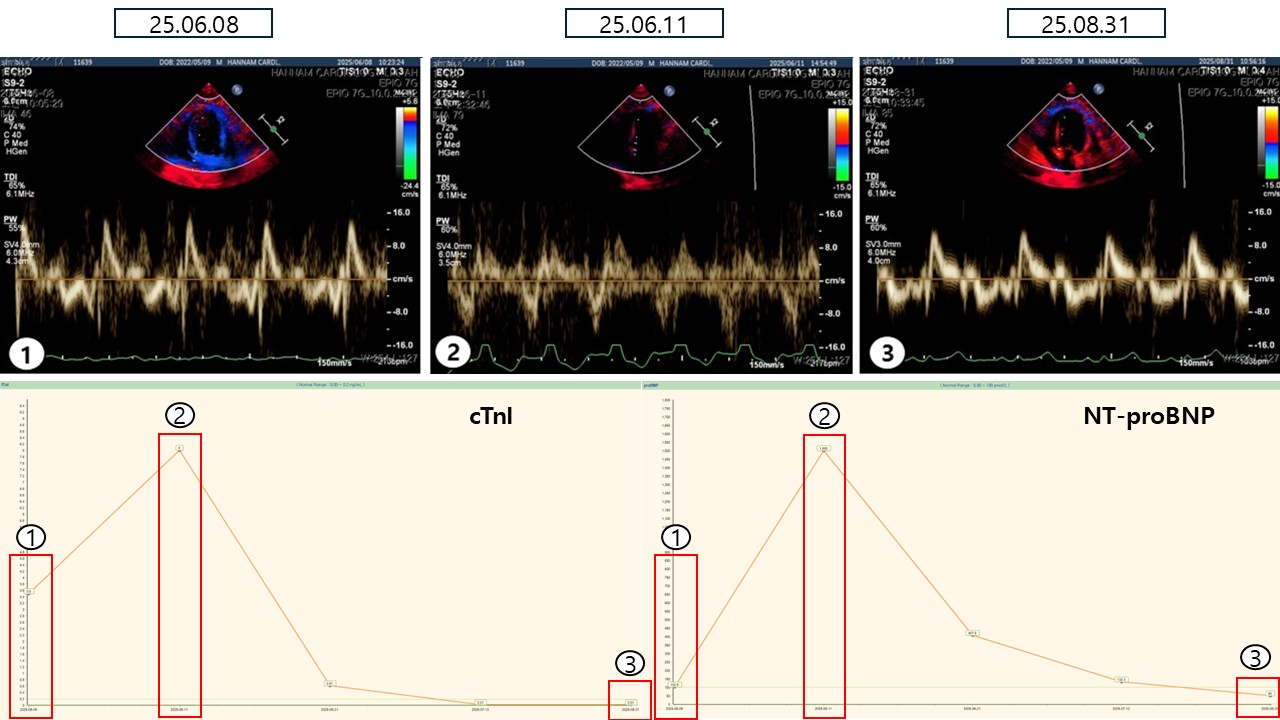

Supplement: Supplementary file 5 [file Image_5.jpeg]
